# Ethyl Acetate Fraction of *Hedyotis diffusa Willd* Induces Apoptosis via JNK/Nur77 Pathway in Hepatocellular Carcinoma Cells

**DOI:** 10.1155/2022/1932777

**Published:** 2022-08-24

**Authors:** Weimin Ning, Nishan Xu, Chunhong Zhou, Lifang Zou, Jingyu Quan, Hua Yang, Zinbin Lu, Huihui Cao, Junshan Liu

**Affiliations:** ^1^Dongguan Hospital of Chinese Medicine affiliated to Guangzhou University of Chinese Medicine, Dongguan 523005, China; ^2^Traditional Chinese Pharmacological Laboratory, Third Level Research Laboratory of State Administration of Traditional Chinese Medicine, School of Traditional Chinese Medicine, Southern Medical University, Guangzhou 510515, China; ^3^Department of Pharmacy, Zhujiang Hospital, Southern Medical University, Guangzhou 510515, China

## Abstract

**Background:**

Hepatocellular carcinoma (HCC) is characterized by poor diagnosis and high mortality. Novel and efficient therapeutic agents are urgently needed for the treatment. *Hedyotis diffusa Willd* (HDW) is used to treat cancers, especially HCC in China.

**Purpose:**

The study aimed to identify the main anti-HCC extract in HDW and to explore the mechanism of the active extract.

**Materials and Methods:**

The high-performance liquid chromatography-quadrupole-time of flight mass spectrometry (HPLC-QTOF-MS) method was used for the simultaneous determination of main compounds in the ethyl acetate fraction of HDW (EHDW). The toxicity test of different HDW fractions was carried out on larvae at 2 day-post-fertilization (dpf) for 72 h. The in vivo anti-HCC effect of different HDW fractions was evaluated on a zebrafish tumor model by immersion administration. The antiproliferative effect of HDW fractions was determined with MTT assay, as well as hematoxylin and eosin (HE) staining assay. Hoechst 33258 staining was used to observe changes in nucleus morphology. Flow cytometry analysis was used to investigate apoptosis induction. Western blot analysis was used to examine apoptosis-related proteins, and key proteins in JNK/Nur77 signaling pathway. SP600125 was served to validate the apoptotic mechanism.

**Results:**

EHDW showed the strongest tumor cell growth inhibitory effect on zebrafish tumor model. Further study revealed that EHDW induced apoptosis in zebrafish tumor model and in cultured Hep3B cells. Meanwhile, it has been shown that the levels of BCL2-associated X (Bax), cytochrome c (cyto c), cleaved-caspase 3, and poly-ADP-ribose polymerase (PARP) cells were upregulated. In contrast, the level of antiapoptotic B cell lymphoma-2 (Bcl-2) was downregulated in Hep3B cells. Additionally, EHDW activated JNK/Nur77 pathway by increasing the levels of p-JNK^(Thr183/Tyr185)^ and p-Nur77^(Ser351)^. Further study showed that blockage of JNK by SP600125 reversed EHDW-induced JNK/Nur77 pathway and the downstream apoptotic proteins.

**Conclusion:**

In conclusion, EHDW exerted the anti-HCC effect, which may be attributed to the activation of JNK/Nur77 pathway. This study supported the rationale of HDW as an HCC therapeutic agent.

## 1. Introduction

As the most primary malignant tumor of the liver and the third leading cause of cancer-related deaths, hepatocellular carcinoma (HCC) remains a public health concern worldwide [1,2]. Ordinarily, two main treatment options for HCC patients are chemotherapy and surgical management. However, though hepatic resection provides a great possibility of tumor cure and control, only almost 30% of patients with HCC meet the requirement for surgery [3]. What's more, the recurrence rate of HCC patients after effective treatment is as high as 70% within 5 years [3]. Taken together, low cure rate and high recurrence rate present an increasing cause of high mortality and poor prognosis in HCC patients. Consequently, it is imperative to find an effective anti-HCC agent.

Cancer can be viewed as the result of the transformation of a normal cell into a malignant one, while evasion of cell death is one of the essential changes in a cell that cause this malignant transformation [4]. Previous researches had linked apoptosis to the elimination of malignant cells and tumor progression. Recent studies have found that apoptosis can affect the physiological process of neighboring cells in the microenvironment through direct contact or release of a series of regulatory factors, which plays an important role in the construction of tumor microenvironment, besides, apoptosis regulates the secretion of inhibitory cytokines such as transforming growth factor-*β*1, thereby inducing immunosuppression or tumor immune tolerance [5]. Therefore, every defect or abnormality along apoptotic pathways may also be an interesting target for cancer treatment. Jun N-terminal kinase (JNK), a member of mitogen-activated protein kinases, is an important signaling component, which converts external stimuli into a wide range of cellular responses, including proliferation, migration, invasion, and apoptosis [6]. Although some studies depicted JNK as a promotor for HCC, it is surprising that mice deficient in JNK in hepatocytes were susceptible to HCC [7]. Furthermore, it has been reported that JNK1 was required for hepatocyte death in the diethylnitrosamine model of HCC [8]. Besides, the activation of the JNK signaling pathway significantly increases the expression of caspase 3 in HCC cells, suggesting that JNK promotes apoptosis in HCC cells [9]. Collectively, all of these data indicated that JNK is closely related to the occurrence and development of HCC. JNK is normally distributed in the cytoplasm, when activated by upstream signals or stimulators, JNK will quickly translocate into the nucleus and phosphorylate a variety of targeted proteins, including Nur77 [10]. Phosphorylated Nur77 has been reported to inhibit the mitogenic effect as well as to promote its localization into mitochondria through interaction with Bcl-2 [11]. Besides, clinical data indicate that Nur77 is a suppressor of HCC. The Oncomine database shows that the gene level of Nur77 in HCC tissues was significantly lower than that in normal liver tissues [12]. Analysis from clinical samples demonstrated that Nur77 expression was decreased with the development of HCC from stage I to III [13]. Moreover, patients with low Nur77 levels had a poor prognosis [13]. Taken together, these results indicated that the decrease of Nur77 facilitates human HCC development, and the JNK/Nur77 pathway may be a potential target for HCC treatment [14–17].

*Hedyotis diffusa Willd* (HDW), a Chinese medicinal herbal, has been widely used as an adjuvant therapeutic agent against hepatoma for decades in China [18]. Recent studies suggested that HDW exhibited anticancer activity with manifestations of apoptosis induction and immune function enhancement [19–21]. However, the benefit from different extracts of HDW for HCC is still unclear, and there is a lack of cognition on the anticancer mechanism of HDW. Herein, we performed the activity screening of HDW extracts and further mechanistic study in vivo and in vitro.

## 2. Materials and Methods

### 2.1. Materials and Reagents

Hoechst 33258 dye was from Thermo Fisher Scientific (Waltham, MA, USA). Annexin V-FITC/PI apoptosis kit was purchased from Invitrogen (Carlsbad, CA, USA). Phosphate buffer saline (PBS) and 3-(4,5-dimethylthiazol-2-yl)-2,5-diphenyltetrazolium bromide (MTT) were obtained from Sigma (St Louis, MO, USA). SP600125 was from Selleck Chemicals (Houston, Texas, USA). All antibodies were purchased from Cell Signaling Technology (Beverly, MA, USA).

### 2.2. Sample Extraction and HPLC-QTOF-MS Analysis

HDW purchased from Kangmei pharmacy (Lot no. 20030011, Guangzhou, China) was identified by Professor Hongwei Zhang (Guangzhou, Southern Medical University) and then subjected to heat reflux with 85%, 75%, and 65% ethanol. After being filtered three times, the combined ethanolic filtrate was concentrated using a rotary evaporator to give a crude extract. Subsequently, the total extract was added with water to make a suspension, which was successively partitioned with solvents of increasing polarity to obtain PHDW (petroleum ether fraction of HDW), EHDW, NHDW (n-butanol fraction of HDW), and HHDW (H_2_O fraction of HDW). Finally, the extracts were dried in a lyophilizer and kept at 20°C until further use.

The HPLC-QTOF-MS method was applied to identify the compositions of EHDW. The analysis was conducted using an HPLC system (AB Sciex ExionLC) equipped with an AB sciex ×500 R QTOF system integrating a switchable electrospray ion source interface (ESI). The mobile phases were 0.1% aqueous formic acid (A) and acetonitrile (B) with a linear gradient elution: 0–5 min, 15% B, 5–10 min, 15–45% B, 10–16 min, 45–80% B, 16–20 min, 80–100% B, 20–25 min, 100% B, 25–40 min, 100–5% B on a silica column (1.8 *μ*m, 2.1 × 100 mm) at a flow rate of 0.3 mL/min. The complete scan was performed in both ESI positive and negative ionization modes in the range of m/z 50–1000 Da. With the help of SCIEX OS 1.7.0 software, we speculated the possible fragmentation pathway of compositions and matched them with the Natural Products HR-MS/MS 2.0 database.

### 2.3. Cell Culture

Hep3B cells were obtained from American Type Culture Collection (ATCC, Rockville, MD, USA). The cells were maintained in RPMI-1640 medium supplemented with 10% (*v/v*) fetal bovine serum (Invitrogen, Grand Island, USA) and 1% penicillin/streptomycin (Invitrogen, Grand Island, USA) at 37°C in a humidified atmosphere with 5% CO_2_.

### 2.4. Cell Viability

Cell viability was measured by MTT assay [22]. Briefly, Hep3B cells (10^4^/well) were seeded in a 96-well plate for 24 h and subsequently exposed to EHDW (50–400 *μ*g/mL) for another 24 h. After being washed with PBS twice, the cells received an incubation with 20 *μ*L of MTT (5 *μ*g/mL) for a further 4 h while avoiding light, and thereafter 100 *μ*L of dimethyl sulfoxide (DMSO, Sigma-Aldrich, St Louis, MO, USA) was added. Finally, the absorbance (A) was detected at 570 nm using a microplate reader (Thermo Fisher Scientific, Waltham, USA). Cell viability was calculated according to the following formula: cell viability = A_test_/A_blank_ × 100%. Furthermore, IC_50_ value was calculated using GraphPad Prism software.

### 2.5. Hoechst 33258 Staining

Hoechst 33258 staining was used to observe the appearance of apoptosis. Hep3B cells were seeded in a six-well plate and then treated with EHDW for 24 h. After being washed with PBS, the cells were stained with Hoechst 33258 at 37°C for 15 min in dark and then were observed under a fluorescent inverted microscope (BX61W1, OLYMPUS, Japan).

### 2.6. Annexin V-FITC/PI Staining Assay

Annexin V-FITC and PI double staining was carried out to discriminate different stages of apoptosis and/or necrosis. In brief, untreated and treated Hep3B cells were trypsinized, centrifuged, and harvested. Thereafter, the collected cells were resuspended in 500 *μ*L of binding buffer containing Annexin V-FITC (1%) and PI (1%) dyes while protected from light. Then, a 15-min incubation at 25°C was given. In the end, flow cytometry analysis (CytoFLEX, Backman Counter, CA, USA) was performed to assess apoptosis induction.

### 2.7. Western Blot Analysis

Hep3B cells were treated with EHDW or SP600125 for 24 h. Then, the harvested cells were lysed on ice for 15 min in RIPA buffer containing 0.1 mM phenylmethylsulfonyl fluoride, 0.1 mM sodium orthovanadate, 0.1 mM dithiothreitol, and phosphatase inhibitor, followed by centrifugation at 13,000 rpm at 4°C for 15 min. The protein concentration was quantitated with a bicinchoninic acid protein assay kit (Thermo Fisher Scientific, Waltham, MA, USA). Subsequent gel electrophoresis and immunoblotting were performed as reported earlier [22].

### 2.8. Toxicity Test on Zebrafish

Nontoxic dosage of HDW in zebrafish was determined by the following toxicity test. In brief, embryos were collected after fertilization and were transferred into a culture dish filled with egg water containing 0.002% methylene blue at 28.5°C for 48 h, and then 30 larvae were exposed to HDW ranging from 0 to 1000 *μ*g/mL by immersion administration. The mortality and malformation of zebrafish were recorded after 3 days.

### 2.9. Microinjection of Zebrafish and Drug Treatment

The collected Hep3B cells were incubated with 2–5 *μ*M CM-Dil for 30 min at 37°C and a further 15 min at 4°C. Next, the cells were resuspended with 0.4% carboxymethyl cellulose-Na and placed on ice until use. Larvae at 2 dpf were yolk-microinjected with 200 stained cells, and then exposed to HDW for 3 days, while sorafenib was prepared as the positive group (*n* = 30). Then, zebrafish were photographed by fluorescence microscopy (Olympus, MVX10, Tokyo, Japan).

### 2.10. Histopathological Analysis of Zebrafish

The treated larvae at 5 dpf were fixed with paraformaldehyde overnight. Further successive dehydration of zebrafish was performed with increasing ethanol and then embedded in paraffin. After being cut into 5-*μ*m thick slides, larvae specimens were stained with hematoxylin and eosin for detection of pathological changes in the yolk sac under an IX 53 light microscope (Olympus, Tokyo, Japan).

### 2.11. Statistical Analysis

The average value with SEM was presented corresponding to 3 or more replicates. One-way analysis of variance (ANOVA) was performed to analyze statistical differences at *P* < 0.05 using GraphPad Prism 5.0 (San Diego, CA, USA).

## 3. Results

### 3.1. Anti-HCC Activity of HDW in a Zebrafish Tumor Model

**Figure 1:**
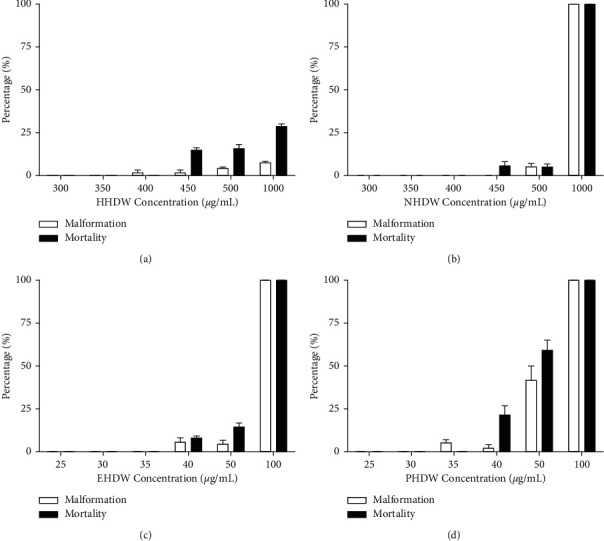
Toxicity evaluation of HDW in zebrafish. 2 dpf larvae were randomly divided into 6 groups (*n* = 30) and immersed with HHDW (a), NHDW (b), EHDW (c), and PHDW (d), respectively. Mortality and malformation were monitored after 72 h.

To investigate the potential toxicity of four extracts from HDW, zebrafish larvae were treated with different concentrations of HHDW, NHDW, EHDW, and PHDW, and then the morphological change and mortality of these zebrafish were monitored after 72 h. As illustrated in Figure 1, the toxicity of HDW extracts increased with the decrease of solvent polarity. Under 100 *μ*g/mL, no abnormality or death was observed in HHDW and NHDW treated zebrafish (data not shown), while zebrafish in EHDW and PHDW groups were all dead. The toxicity of HDW fractions was PHDW > EHDW > NHDW > HHDW. The nontoxic concentration was HHDW ≤ 350 *μ*g/mL, NHDW ≤ 400 *μ*g/mL, EHDW ≤ 35 *μ*g/mL, and PHDW ≤ 30 *μ*g/mL. Under these concentrations, the extractions did not show any toxic effects in zebrafish, including yolk sac rupture, malformation, and death. Then, we selected three concentration gradients for further study: 300, 200, and 100 *μ*g/mL for HHDW and NHDW, and 30, 20, and 10 *μ*g/mL for EHDW and PHDW.

**Figure 2:**
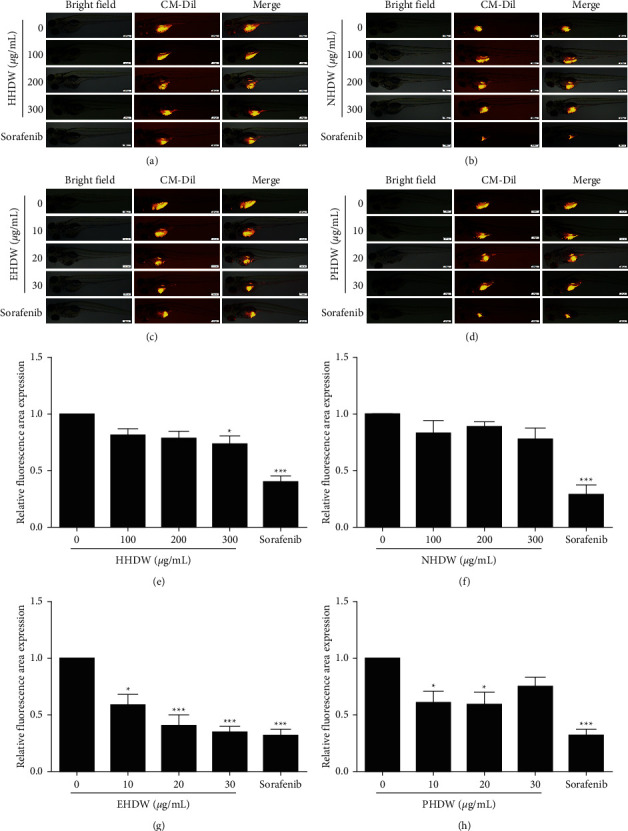
Antitumor activity of different extracts of HDW in zebrafish xenograft model. EHDW inhibited the proliferation of Hep3B cells in vivo. Hep3B cell-bearing zebrafish were treated with HHDW (a), NHDW (b), EHDW (c), PHDW (d), or sorafenib (0.25 *μ*M) for 72 h and then CM-Dil-labeled Hep3B cells in yolk sac were visualized using a fluorescence microscope. Left: bright field, middle: CM-Dil-staining cells, right: merge. Bar: 200 *μ*m. (e–h). Quantitative analysis of the fluorescent area in zebrafish. ^*∗*^*P* < 0.05, ^*∗∗*^*P* < 0.01, and ^*∗∗∗*^*P* < 0.001 vs. control.

**Figure 3:**
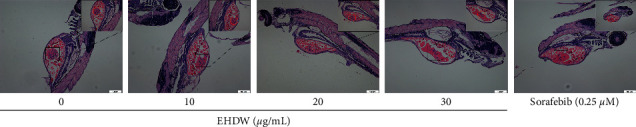
EHDW inhibited the tumorigenicity of Hep3B cells in zebrafish. Larvae were microinjected with 200 cells, and then treated with EHDW at 10, 20, and 30 *μ*g/mL or sorafenib (0.25 *μ*M). After 72 h, zebrafish were dehydrated and embedded in paraffin for HE staining. Bar: 50 *μ*m. Red circles indicated tumor cells.

Then, to determine whether the four extractions have antitumor activity, a zebrafish tumor model was established. The antitumor effect of HDW extracts was evaluated by the intensity of red fluorescence. As shown in Figure 2(a), after treatment, the intensity and area of red fluorescence decreased notably in a dose-dependent manner in EHDW group, and a similar phenomenon was observed after sorafenib treatment. However, the fluorescence has not changed obviously in HHDW (Figure 2(a)), NHDW (Figure 2(b)), and PHDW groups (Figure 2(d)). As shown in Figure 3, a large number of tumor cells were observed in the yolk sac in the control group, while the numbers of Hep3B cells decreased after EHDW treatment. HE staining data demonstrated that cell proliferation and tumor formation in the EHDW-treated group were significantly inhibited compared with the control group. In view of the above results, EHDW was selected as the research object.

Firstly, HPLC-QTOF-MS analysis was applied to identify compounds in EHDW. The total ion chromatograms were shown in Figure S1. Based on retention time, ion current mass spectrometry, and literature reports, a total of 55 compounds from EHDW were confirmed, including anthraquinones, flavonoids, and organic acids (Table S1).

### 3.2. EHDW Inhibited Hep3B Cell Proliferation

**Figure 4:**
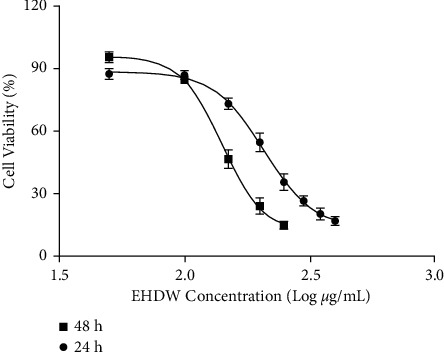
EHDW exerted cytotoxicities in Hep3B cells in dose- and time-dependent manners. Hep3B cells were treated with various concentrations of EHDW for 24 and 48 h MTT assay was used to evaluate cell viability.

To determine the cytotoxicity of EHDW in vitro, we conducted MTT assay in Hep3B cells after EHDW treatment for 24 and 48 h, respectively. As shown in Figure 4, EHDW significantly decreased the viability of Hep3B cells dose- and time-dependently, IC_50_ value for 24 h was 200 *μ*g/mL, IC_50_ value for 48 h was 150 *μ*g/mL. Therefore, we used 150, 200, and 250 *μ*g/mL of EHDW in subsequent cell experiments.

### 3.3. EHDW Induced Hep3B Cell Apoptosis In Vitro and In Vivo

**Figure 5:**
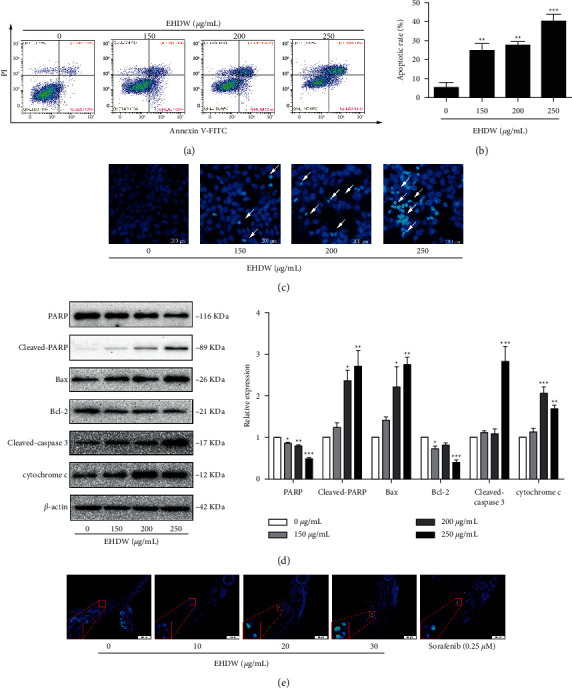
EHDW induced apoptosis in Hep3B cells. (a) Cell apoptosis was measured using flow cytometry. Representative data were analyzed with Annexin-V/PI staining in Hep3B cells after 24 h of exposure to indicated concentrations of EHDW. (b) Flow cytometry analysis of the percentage of apoptotic cells (early apoptosis and late apoptosis) from three independent experiments. ^*∗∗*^*P* < 0.01 and ^*∗∗∗*^*P* < 0.001 vs. control. (c) Apoptotic morphological observation. Fluorescence microscope images showed morphological changes in Hep3B cells upon 150–250 *μ*g/mL EHDW treatment for 24 h White arrows showing bright blue regions indicated nuclear pyknosis and fragmentation of chromatin. (d) Expression of cyto c in cytosol and PARP, cleaved-PARP, Bcl-2, BAX, cleaved-caspase 3 in total proteins were examined by Western blotting. ^*∗*^*P* < 0.05, ^*∗∗*^*P* < 0.01, and ^*∗∗∗*^*P* < 0.001 vs. control. One-way ANOVA, post hoc comparisons, Tukey's test. Columns, means, error bars, SEM. (e) EHDW induced apoptosis in vivo. The 5-*μ*m zebrafish tissue sections were deparaffinized and stained with Hoechst 33258 dye. Representative fluorescent photographs were taken by fluorescence microscopy.

To investigate whether EHDW can induce cell death by apoptosis, Annexin V-FITC/PI double assay was applied to detect the translocation of phosphatidylserine residues on the plasma membrane. Cytometric results (Figures 5(a) and 5(b)) indicated that the percentage of apoptotic cells (early and late apoptosis) increased from 5.51% in untreated cells to 26.92% (150 *μ*g/mL), 29.82% (200 *μ*g/mL), and 43.3% (250 *μ*g/mL) in treated cells. Hoechst 33258 staining was further employed to differentiate between the apoptotic and normal cells. As shown in Figure 5(c), the control group displayed normal nuclear morphology with uniform diffuse fluorescence. In contrast, treated cells showed distinct apoptotic nuclear morphology with a condensed bright blue nucleus in a concentration-dependent manner. These data confirmed that EHDW induced apoptotic cell death.

To further corroborate the results of morphological analysis and flow cytometry, PARP, which is associated with DNA damage repair and is also an important marker for apoptosis, was detected. As demonstrated in Figure 5(d), the immunoblotting analysis showed that EHDW treatment resulted in a decreased expression of PARP and the activation of cleaved-PARP. It is well known that the Bcl-2 protein family and caspase family play an important role in the regulation of cell apoptosis. To study whether EHDW-induced apoptosis is associated with the activation of intrinsic apoptotic pathways, we further analyzed activities and levels of apoptosis-related proteins in Hep3B cells in response to EHDW. As seen in Figure 5(d), treatment with EHDW enhanced Bax protein expression, and reduced Bcl-2 protein expression, thus the Bax/Bcl-2 ratio was increased. Meanwhile, cleaved-caspase 3 was increased as well.

Subsequently, to determine if EHDW can induce apoptosis in the zebrafish tumor model, Hoechst 33258 assay was applied to observe nuclear changes in zebrafish tumor tissues. As shown in Figure 5(e), EHDW and sorafenib facilitated apoptosis of tumor cells in transplanted zebrafish as evidenced by chromatin condensation in the treated groups compared with that in the control group. The results further confirmed that the antitumor activity of EHDW in the transplanted tumor was associated with apoptosis.

### 3.4. EHDW Induced Apoptosis through JNK/Nur77 Pathway

**Figure 6:**
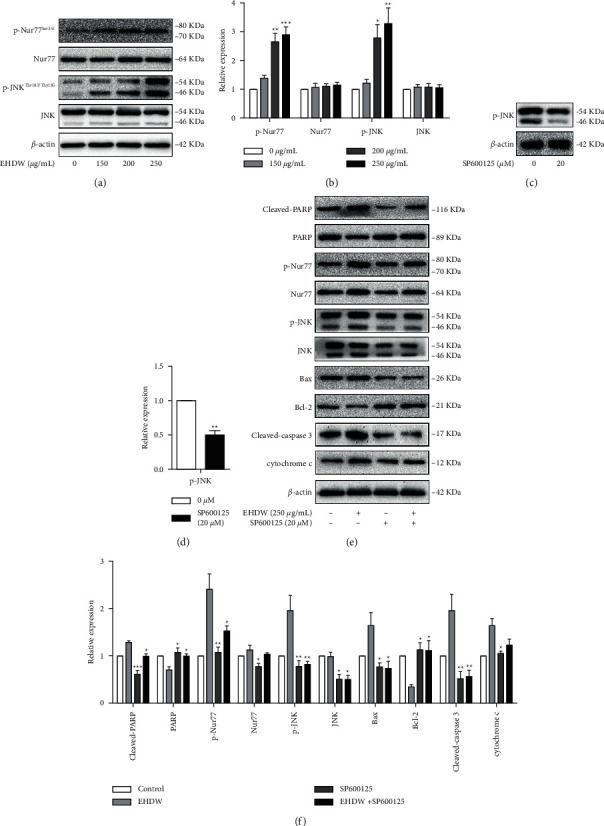
EHDW induced apoptosis in Hep3B cells by JNK/Nur77 pathway. (a–b) EHDW activated JNK/Nur77 pathway. After incubation for 24 h, the protein levels of JNK, p-JNK^(Thr183/Tyr185)^, Nur77, and p-Nur77^(Ser351)^ were measured by Western blotting. ^*∗*^*P* < 0.05, ^*∗∗*^*P* < 0.01, and ^*∗∗∗*^*P* < 0.001 vs. control. One-way ANOVA, post hoc comparisons, Tukey's test. Columns, means, error bars, SEM. (c–d) The functional verification of SP600125. Hep3B cells were treated with SP600125 for 24 h Western blotting was performed to detect the expression of p-JNK. ^*∗*^*P* < 0.05, ^*∗∗*^*P* < 0.01, and ^*∗∗∗*^*P* < 0.001 vs. control. One-way ANOVA, post hoc comparisons, Tukey's test. Columns, means, error bars, SEM. (e–f) The apoptotic effect of EHDW were counteracted in the presence of SP600125. The expression of proteins in JNK/Nur77 pathway and the downstream-related proteins were detected after the cells were treated with SP600125 and EHDW for 24 h. ^*∗*^*P* < 0.05, ^*∗∗*^*P* < 0.01, and ^*∗∗∗*^*P* < 0.001 vs. EHDW treatment. One-way ANOVA, post hoc comparisons, Tukey's test. Columns, means, error bars, and SEM.

It is well known that Nur77 possesses both oncogenic and tumor suppressor activities [23–25]. In response to apoptotic stimuli, JNK regulates positively Nur77 translocating from nucleus to cytoplasm where it targets mitochondria to induce apoptosis in cancer cells. To further explore the mechanism of EHDW-induced apoptosis, the expression levels of JNK, p-JNK^(Thr183/Tyr185)^, Nur77, and p-Nur77^(Ser351)^ were investigated by Western blotting. As shown in Figure 6(a), EHDW dose-dependently increased the levels of p-JNK^(Thr183/Tyr185)^ and p-Nur77^(Ser351)^. These results implied that EHDW induced apoptosis through JNK/Nur77 signaling.

To further clarify whether JNK activation was dominant in EHDW-induced apoptosis, the effects of JNK inhibitor SP600125 on the apoptosis of Hep3B cells treated with EHDW were examined. As shown in Figure 6, following the addition of 20 *μ*M SP600125 in HCC cells, the phosphorylation of JNK induced by EHDW was suppressed followed by the inhibition of its downstream Nur77. Moreover, SP600125 treatment blocked the downregulation of Bcl-2 as well as resulted in the decrease of Bax, cleaved‐caspase 3, and cleaved‐PARP. These results indicated the activation of JNK/Nur77 pathway played a critical role in EHDW‐induced caspase‐dependent apoptosis in HCC cells. However, further research is still needed to identify the role of this pathway in EHDW-induced apoptosis.

## 4. Discussion

HDW is used for treating cancer in clinic [18]. However, the effect of HDW on HCC and the underlying mechanisms have not been fully explored. Zebrafish in vivo model is an attractive tool with the advantages of high reproduction rate, low cost, high genetic similarity to human, and easy of genetic manipulation of embryos by microinjection. Moreover, the transparency of embryo allows dynamic observation of tumor-related processes such as cell proliferation, cell invasion, and cell metastasis in vivo. Therefore, zebrafish xenograft model facilitates large-scale screening with the goal of identifying new anticancer candidates [26–28]. In the present study, we first established a zebrafish tumor model to evaluate the antitumor activity of different polar extracts of HDW and then found that EHDW exerted the most significantly inhibitory effects on Hep3B cells. Furthermore, we demonstrated that EHDW induced apoptosis in Hep3B cells by activating JNK/Nur77 signaling pathway.

Apoptosis is generally confirmed by visible morphological characteristics and biochemical markers [29]. Usually, the process of apoptosis involves nuclear membrane breakdown and DNA fragmentation. Pyknosis is the result of chromatin condensation, which is the most prominent feature of apoptosis [30]. Biochemical markers of apoptosis include exposure of phosphatidylserine on the cell surface and the activation of proteases termed caspases such as caspase 3. A critical protein marker for caspase 3 activation is the appearance of the cleavage of PARP. In the present study, we reported that EHDW reduced cell viability and inhibited the growth of Hep3B cells dose- and time-dependently, implying its anti-HCC potential. Furthermore, we demonstrated that EHDW induced apoptosis in Hep3B cells as evidenced by phosphatidylserine translocation, nuclear condensation, and PARP cleavage. Moreover, under cellular stress response, the intrinsic apoptotic pathway can be activated by the stimulation of a variety of mitochondrial molecular proteins, such as Bcl-2, cyto *c*, and caspases [31–33]. The increased of Bax/Bcl-2 ratio leads to the decrease of mitochondrial membrane potential, which further promotes the release of cyto *c* from mitochondria, thereby activating caspases. Our study showed that EHDW induced apoptosis in HCC cells, which was accompanied by the increase of Bax/Bcl-2 ratio, the release of cyto *c* in cytosol, as well as the increased cleavages of caspase 3 and PARP. Therefore, EHDW-induced apoptosis may be mediated by the intrinsic apoptosis pathway.

It was reported that JNK was rapidly activated in apoptotic cells in response to diverse stimuli, such as oxidative stress, endoplasmic reticulum stress, DNA damage, or metabolic changes, and the overexpression of JNK could induce apoptosis in HCC cells [34]. The activation of JNK can phosphorylate Nur77, leading to Nur77 translocation from nuclear to mitochondria. In cancer cells, Nur77 functions in nucleus as a survival factor, but becomes a potent killer when certain death stimuli induce its migration to mitochondria, where it binds to Bcl-2 and conformationally converts it to a killer that triggers cyto *c* release and caspase activation [17,35]. Collectively, the JNK/Nur77 signaling pathway may be a target for tumor therapy. Interestingly, we found that EHDW increased the expression of p-JNK^(Thr183/Tyr185)^ and p-Nur77^(Ser351)^. To verify EHDW-induced apoptosis was indeed mediated by JNK/Nur77 activation, we investigated whether SP600125 could abrogate EHDW-mediated apoptosis. Our results revealed that the inhibition of JNK with SP600125 significantly reversed EHDW-induced phosphorylation of Nur77 at Ser351, and then attenuated the increase in Bax/Bcl-2 ratio, cyto *c* release, caspase 3 activation, and PARP cleavage in response to EHDW. Taken together, we illustrate that EHDW-triggered apoptosis of HCC cells may be related to the activation of JNK/Nur77 signaling pathway.

## 5. Conclusion

Our research first evaluated the antitumor activity of different extracts of HDW, and found that EHDW with most significant effect induced apoptosis through the activation of JNK/Nur77 pathway. These findings provided a research basis for HDW as an effective drug for the treatment of HCC.

## Data Availability

The data used to support the findings of this study are included within the article.

## Supplementary Materials

Supplementary Materials Figure S1. Total ion chromatogram of EHDW in positive (A) and negative (B) ion mode. Table S1. Identification of chemical constituents of EHDW.
