# Supplementary material for: Ethyl Acetate Fraction of Hedyotis diffusa Willd Induces Apoptosis via JNK/Nur77 Pathway in Hepatocellular Carcinoma Cells
Source: Evid Based Complement Alternat Med. 2022 Aug 24;2022:1932777. doi: 10.1155/2022/1932777 (PMC9433286; doi:10.1155/2022/1932777)
Supplement: Supplementary Materials — Figure S1. Total ion chromatogram of EHDW in positive (A) and negative (B) ion mode. Table S1. Identification of chemical constituents of EHDW. [file 1932777.f1.docx]

**Supplementary materials**

**
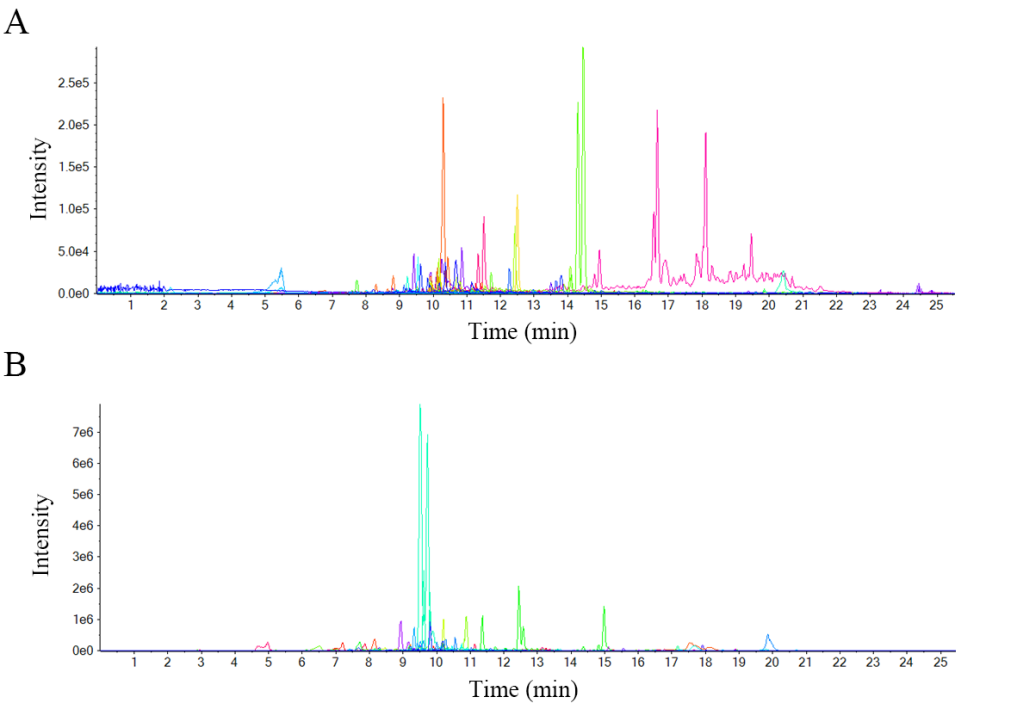
**

Fig.S1 Total ion chromatogram of EHDW in positive (A) and negative (B) ion mode.

Tab.S1. Identification of chemical constituents of EHDW.

| NO. | RT (min) | Molecular formula | Molecular ion | *m/z* | Identified compounds | Library score |
| --- | --- | --- | --- | --- | --- | --- |
| 1 | 9.62 | C_21_H_20_O_11_ | [M+H]^+^ | 449.1059 | Quercitrin | 100.0 |
| 2 | 5.47 | C_10_H_13_N_5_O_4_ | [M+H]^+^ | 268.1034 | Adenosine | 100.0 |
| 3 | 9.21 | C_10_H_8_O_4_ | [M-H]^-^ | 191.0341 | Isoscopoletin | 100.0 |
| 4 | 10.19 | C_7_H_6_O_3_ | [M-H]^-^ | 137.0240 | 4-Hydroxybenzoic acid | 100.0 |
| 5 | 8.93 | C_9_H_8_O_3_ | [M-H]^-^ | 163.0394 | p-Coumaric acid | 99.7 |
| 6 | 10.29 | C_10_H_8_O_4_ | [M+H]^+^ | 193.0494 | Scopoletin | 99.4 |
| 7 | 11.21 | C_11_H_12_O_4_ | [M-H]^-^ | 207.0656 | Ethyl Caffeate | 99.4 |
| 8 | 10.19 | C_7_H_6_O_3_ | [M-H]^-^ | 137.0240 | Salicylic acid | 99.3 |
| 9 | 7.73 | C_17_H_24_O_11_NH_3_ | [M+NH_3_]^+^ | 422.1639 | Deacetyl asperulosidic acid methyl ester | 99.0 |
| 10 | 12.51 | C_16_H_12_O_7_ | [M+H]^+^ | 317.0646 | Isorhamnetin | 99.0 |
| 11 | 20.43 | C_15_H_24_ | [M+H]^+^ | 205.1947 | Patchouli alcohol | 98.9 |
| 12 | 9.21 | C_10_H_8_O_4_ | [M-H]^-^ | 191.0341 | Isoscopoletin | 98.9 |
| 13 | 11.26 | C_20_H_17_NO_4_ | [M+H]^+^ | 336.1223 | Berberine | 98.7 |
| 24 | 9.62 | C_21_H_20_O_11_ | [M+H]^+^ | 449.1059 | Quercetin 7-rhamnoside | 98.7 |
| 15 | 9.62 | C_21_H_20_O_11_ | [M+H]^+^ | 449.1059 | Rhodionin | 98.7 |
| 16 | 7.68 | C_8_H_8_O_4_ | [M-H]^-^ | 167.0342 | Vanillic acid | 98.6 |
| 17 | 7.73 | C_17_H_24_O_11_NH_3_ | [M+NH_3_]^+^ | 422.1639 | Gardenoside | 98.5 |
| 18 | 9.40 | C_21_H_20_O_11_ | [M-H]^-^ | 447.0911 | Homoorientin (Isoorientin) | 98.4 |
| 19 | 9.40 | C_21_H_20_O_11_ | [M-H]^-^ | 447.0911 | Astragalin | 98.4 |
| 20 | 10.19 | C_9_H_10_O_4_ | [M+H]^+^ | 183.0651 | Syringaldehyde | 98.3 |
| 21 | 13.51 | C_14_H_8_O_4_ | [M+H]^+^ | 241.0492 | 1,8-Dihydroxyanthraquinone | 97.7 |
| 22 | 9.93 | C_27_H_30_O_15_ | [M+H]^+^ | 595.1633 | Aempferol-3-O-rutinoside | 97.6 |
| 23 | 9.55 | C_10_H_8_O_5_ | [M+H]^+^ | 209.0443 | Fraxetin | 97.4 |
| 24 | 8.15 | C_7_H_6_O_2_ | [M-H]^-^ | 121.0292 | 4-Hydroxybenzaldehyde | 96.7 |
| 25 | 19.86 | C_15_H_22_O | [M+H]^+^ | 219.1738 | Germacrone; | 96.4 |
| 26 | 7.41 | C_18_H22O_11_ | [M-H]^-^ | 413.1073 | Asperuloside | 96.4 |
| 27 | 9.23 | C_10_H_10_O_4_ | [M-H]^-^ | 193.0497 | Isoferulic acid | 96.3 |
| 28 | 2.95 | C_16_H_22_O_11_ | [M-H]^-^ | 389.1074 | 10-Deacetylasperulosidic acid | 95.9 |
| 29 | 9.23 | C_10_H_10_O_4_ | [M-H]^-^ | 193.0497 | Ferulic Acid | 95.9 |
| 31 | 2.20 | C_5_H_5_N_5_ | [M+H]^+^ | 136.0620 | Adenine | 95.1 |
| 32 | 9.23 | C_9_H_6_O_4_ | [M+H]^+^ | 179.0340 | Daphnetin | 95.0 |
| 32 | 10.67 | C_11_H_10_O_5_ | [M+H]^+^ | 223.0600 | 6-hydroxy-7,8-dimethoxycoumarin | 94.6 |
| 33 | 10.68 | C_11_H_10_O5 | [M+H]^+^ | 223.0600 | 8-hydroxy-6,7-dimethoxycoumarin | 94.6 |
| 34 | 7.70 | C_9_H_6_O_4_ | [M-H]^-^ | 177.0185 | Esculetin | 94.2 |
| 35 | 10.68 | C_11_H_10_O_5_ | [M+H]^+^ | 223.0600 | Isofraxidin | 92.8 |
| 36 | 7.20 | C_7_H_12_O_6_ | [M-H]^-^ | 191.0553 | D-(-)-Quinic acid | 91.2 |
| 37 | 9.40 | C_21_H_20_O_11_ | [M-H]^-^ | 447.0911 | Cyanidin-3-O-glucoside | 89.9 |
| 38 | 7.20 | C_7_H_12_O_6_ | [M-H]^-^ | 191.0553 | Quinic acid | 89.7 |
| 39 | 16.66 | C_18_H_30_O_2_ | [M+H]^+^ | 279.2307 | Linolenic acid | 88.1 |
| 40 | 12.03 | C_17_H_14_O_6_ | [M-H]^-^ | 313.0703 | Ladanein | 87.7 |
| 41 | 8.91 | C_19_H_18_O_11_ | [M+H]^+^ | 423.0910 | Mangiferin | 87.2 |
| 42 | 12.43 | C_15_H_10_O_6_ | [M+H]^+^ | 287.0544 | Kaempferol | 86.8 |
| 43 | 12.03 | C_17_H_14_O_6_ | [M-H]^-^ | 313.0703 | Pectolinarigenin | 86.2 |
| 44 | 14.09 | C_15_H_20_O_2_ | [M+H]^+^ | 233.1531 | Costunolide | 85.5 |
| 45 | 10.86 | C_10_H_8_O_3_ | [M+H]^+^ | 177.0548 | Hymecromone | 84.1 |
| 46 | 4.96 | C_7_H_6_O_4_ | [M-H]^-^ | 153.0188 | Protocatechuic acid | 83.1 |
| 47 | 9.40 | C_21_H_20_O_11_ | [M-H]^-^ | 447.0911 | Luteoloside | 79.1 |
| 48 | 14.09 | C_15_H_20_O_2_ | [M+H]^+^ | 233.1531 | Isoalantolactone | 77.9 |
| 49 | 12.43 | C_15_H_10_O_6_ | [M+H]^+^ | 287.0544 | Luteolin | 77.8 |
| 50 | 7.86 | C_9_H_8_O_4_ | [M-H]^-^ | 179.0342 | Caffeic acid | 77.1 |
| 51 | 9.07 | C_14_H_6_O_8_ | [M-H]^-^ | 300.9978 | Ellagic Acid | 74.8 |
| 52 | 14.48 | C_16_H_12_O_4_ | [M+H]^+^ | 269.0801 | Techtochrysin | 74.6 |
| 53 | 11.51 | C_21_H_20_O_9_ | [M+H]^+^ | 417.1167 | Cleomiscosin C | 74.4 |
| 54 | 10.34 | C_15_H_8_O_6_ | [M-H]^-^ | 283.0235 | Rhein | 73.4 |
| 55 | 12.26 | C_17_H_14_O_7_ | [M+H]^+^ | 331.0803 | Tricin | 71.3 |
